# Clinical evidence on non-viral CAR-T cell therapies for solid tumors: a scoping review

**DOI:** 10.3389/fimmu.2026.1905685

**Published:** 2026-08-20

**Authors:** Favio Varón Suárez, Juan Moreno, Oriana Arias-Valderrama, Juan Baena

**Affiliations:** 1 Fellow in Hematology and Clinical Oncology, Universidad Icesi, Cali, Colombia; 2 Resident in Internal Medicine, Universidad Icesi, Cali, Colombia; 3 Professor and Methodological Tutor, Universidad Icesi, Cali, Colombia; 4 Hematologist-Oncologist, Fundación Valle del Lili, Cali, Colombia; 5 LiliCAR-T Group, Fundación Valle del Lili, ICESI, Cali, Colombia; 6 Center for Cell and Gene Therapy, Baylor College of Medicine, Houston, TX, United States

**Keywords:** chimeric antigen receptor T-cell therapy, mRNA electroporation, non-viral gene delivery, piggyBac transposon, scoping review, solid tumors

## Abstract

**Background:**

Chimeric antigen receptor (CAR) T-cell therapy in solid tumors is hindered by the immunosuppressive tumor microenvironment and by toxicities associated with viral-vector manufacturing. Non-viral gene delivery platforms have emerged as a potential alternative, though clinical evidence remains fragmented.

**Methods:**

Following an *a priori* protocol registered on the Open Science Framework (OSF; https://doi.org/10.17605/OSF.IO/2TPQS) and adhering to JBI/PRISMA-ScR guidelines, a systematic search was conducted across four databases from inception through May 15, 2026. Patient-level data were extracted to describe cellular persistence and clinical outcomes across strictly non-viral delivery platforms.

**Results:**

Four early-phase studies met the inclusion criteria, encompassing 28 heavily pretreated patients with metastatic solid tumors. Two non-viral platforms were identified: mRNA electroporation (n=19; intravenous in 13, intratumoral in 6) and the piggyBac transposon system (n=9). Across both mRNA routes, transient CAR-T persistence (<7 days) was observed, with no objective responses (ORR 0%), though disease stabilization yielded a disease control rate (DCR) of 53%; cross-route comparison is limited by differing distribution profiles. The piggyBac system showed longer persistence (~28 days) and a DCR of 78%, including the only documented objective response (ORR 11%). No Grade ≥3 cytokine release syndrome or neurotoxicity was reported in any of the 28 patients, and no tocilizumab or systemic corticosteroids were required.

**Conclusions:**

Within this limited early-phase evidence base, no severe toxicities attributable to non-viral platforms were reported, and the evidence identifies knowledge gaps warranting prospective investigation. mRNA platforms showed transient persistence and disease stabilization in 53% of patients. One partial response was documented with the piggyBac platform in a single patient; however, this outcome cannot be attributed to the delivery platform given simultaneous differences in target antigen, tumor histology, route of administration, and geographic setting. No firm conclusions regarding comparative platform performance can be drawn from this evidence base.

**Systematic review registration:**

https://doi.org/10.17605/OSF.IO/2TPQS, identifier OSF.IO/2TPQS.

## 1 Introduction

The advent of Chimeric Antigen Receptor (CAR) T-cell therapy has fundamentally transformed the therapeutic algorithm for hematological malignancies, achieving historically high rates of durable remission in patients with relapsed or refractory B-cell leukemias and lymphomas (1). Despite this success, translating these results to solid tumors has remained a formidable challenge. The barriers are multifactorial and include poor T-cell trafficking, limited tumor infiltration imposed by a dense physical stroma, and a profoundly immunosuppressive tumor microenvironment (TME) characterized by metabolic exhaustion and inhibitory signaling (2). Non-viral gene delivery platforms may offer distinct advantages in this context: transient mRNA approaches enable rapid dose-escalation and early safety assessment before commitment to persistent cell therapy, while stable genomic integration via transposons may support the sustained cellular persistence needed to overcome the hostile solid-tumor microenvironment. These mechanistic distinctions, rooted in the biology of solid tumors, underscore the need for rigorous clinical characterization of both non-viral approaches.

The regulatory-approved CAR-T cell manufacturing paradigm currently relies almost exclusively on viral vectors — primarily lentivirus and γ-retrovirus — to achieve stable transgene integration. Although highly efficient, viral delivery carries important logistical and clinical limitations, including prolonged manufacturing times, high production costs under Good Manufacturing Practice (GMP) standards, and inherent genomic risks such as insertional mutagenesis and potential oncogenesis, as historically observed in retroviral gene-therapy trials (3). Moreover, the sustained *in vivo* CAR-T cell expansion and persistent constitutive expression associated with viral vector integration frequently precipitate severe and potentially fatal systemic toxicities, most notably cytokine release syndrome (CRS) and immune effector cell-associated neurotoxicity syndrome (ICANS) (4). These toxicities are primarily attributable to uncontrolled T cell activation and excessive cytokine production *in vivo*—a consequence of CAR-T cell biology and *in vivo* kinetics—rather than to manufacturing-related factors. In contrast, transient non-viral approaches (e.g., mRNA electroporation) limit CAR expression duration and may thereby mitigate prolonged expansion and associated toxicity risks.

To circumvent these limitations, strictly non-viral gene-delivery platforms have emerged as a promising alternative for next-generation CAR-T cell engineering (5). Physical or transposition-based methods — specifically messenger RNA (mRNA) electroporation and the piggyBac transposon system (6, 7) — offer distinct advantages in biosafety, manufacturing scalability, and genomic precision. mRNA-based transfection enables rapid generation of CAR-T cells through transient receptor expression, providing a self-limiting pharmacological profile that may mitigate long-term toxicity risks and is particularly advantageous in early-phase dose-escalation studies. Transposon systems, by contrast, mediate stable genomic integration with a larger cargo capacity and substantially lower production complexity than their viral counterparts, potentially providing more sustained cellular persistence. Both platforms represent distinct, complementary approaches to non-viral gene delivery, each with potential advantages and limitations that require rigorous clinical investigation rather than *a priori* assumptions of superiority. Other non-viral engineering platforms, including the Sleeping Beauty transposon system, have similarly been explored in preclinical and early translational studies, offering complementary advantages such as reduced immunogenicity and an integration efficiency comparable to piggyBac; however, Sleeping Beauty-based constructs were not identified in the present search as meeting full inclusion criteria for peer-reviewed clinical data in solid tumors and therefore fall outside the scope of this review.

### 1.1 Rationale and objectives

Although the preclinical rationale for strictly non-viral CAR-T cells in solid tumors is compelling, the corresponding human clinical evidence remains emerging and highly fragmented. The current literature consists predominantly of early-phase clinical trials and small pilot studies with heterogeneous methodologies (8). A rigorous scoping of this literature is therefore needed to characterize the extent and nature of what is currently known about strictly non-viral approaches in this setting, to identify existing knowledge gaps, and to distinguish this emerging field from hybrid models that retain viral components and thus represent a distinct manufacturing and biological category.

The objective of this scoping review is to systematically describe and map the available clinical evidence on strictly non-viral gene-delivery methods for the generation of CAR-T cells in solid tumors. By charting manufacturing feasibility and the outcomes reported in the primary literature, this review aims to define the current evidence base for mRNA and transposon platforms and to identify priorities for future clinical investigation.

## 2 Methods

### 2.1 Protocol and registration

This scoping review was conducted in accordance with the Joanna Briggs Institute (JBI) methodology for scoping reviews (9). The reporting strictly follows the Preferred Reporting Items for Systematic Reviews and Meta-Analyses extension for Scoping Reviews (PRISMA-ScR) guidelines (10). The study protocol was registered *a priori* on the Open Science Framework (OSF; registration: https://doi.org/10.17605/OSF.IO/2TPQS) to ensure methodological transparency, maintain strict adherence to the intervention criteria, and minimize selection bias throughout the screening process. A completed PRISMA-ScR checklist is provided in **Supplementary Material S1**.

### 2.2 Eligibility criteria

To isolate the true clinical impact of non-viral platforms, studies were selected based on the following predefined inclusion criteria: (i) human subjects with histologically confirmed solid tumors; (ii) intervention involving CAR-T cell therapy generated through strictly non-viral gene delivery platforms (e.g., mRNA electroporation or transposon systems); and (iii) reports providing extractable clinical outcomes related to the therapeutic approach (RECIST criteria, adverse events, or cellular kinetics). We included Phase I/II clinical trials, pilot studies, and formal case series.

Exclusion criteria were strictly defined to eliminate confounding variables and encompassed: (i) preclinical or *in vitro* studies; (ii) reviews, commentaries, conference abstracts, congress poster presentations, or any report lacking complete, peer-reviewed individual patient data; and (iii) studies utilizing viral vectors or any hybrid manufacturing workflows.

### 2.3 Information sources and search strategy

A systematic search strategy was performed across four major electronic databases: PubMed/MEDLINE, Embase, Scopus, and the Web of Science Core Collection, from database inception through May 15, 2026. The search strategy utilized a combination of Medical Subject Headings (MeSH) and relevant free-text keywords, including: “Chimeric Antigen Receptor T-Cells”, “non-viral”, “mRNA”, “electroporation”, “transposon”, “piggyBac”, “gene delivery”, and “solid tumor”. No peer-reviewed publications meeting the predefined inclusion criteria were identified after 2023, reflecting the nascent and limited evidence base for strictly non-viral CAR-T approaches in solid tumors during the 2023–2026 period. Although no language restrictions were imposed during the search strategy development, all reports meeting final inclusion criteria were published in English, reflecting either the absence of non-English publications in this field or unavailability of translations for screening. The search strategy was peer-reviewed by a medical librarian prior to execution to ensure comprehensive coverage across all four databases. No structured grey literature search (e.g., trial registries, dissertations) was conducted beyond these four primary databases, which represents a limitation reflected in the Limitations section. The complete Boolean search strings for each database are provided in **Supplementary Material S2**.

### 2.4 Selection of sources of evidence

Search results were exported to the Rayyan platform for systematic screening (11). Two independent reviewers screened titles and abstracts against the predefined eligibility criteria. Potentially relevant reports were retrieved for full-text evaluation. Discrepancies during the selection process were resolved through consensus or consultation with a senior oncologist. The deliberate exclusion of conference abstracts and poster presentations without associated full-text publications was based on the requirement for complete, peer-reviewed individual patient data to ensure methodological rigor. This decision reflects best practice for systematic scoping reviews but represents a potential limitation in a rapidly emerging field, as significant clinical advances may initially be disseminated through preliminary conference reporting (e.g., the excluded Sun et al., 2025 mesothelioma trial).

### 2.5 Data charting process and data items

Data were extracted at the individual patient level (IPD) whenever available, using a structured extraction matrix comprising 53 clinical and technical variables developed *a priori* to ensure comprehensiveness. Core variables mapped to the review objectives included: study characteristics, patient demographics, prior lines of therapy, primary histology, a detailed binary mapping of metastatic sites, lymphodepletion regimens, delivery platform (*piggyBac* or mRNA), target antigen, CAR-T cell dose, best global response (RECIST 1.1), cellular persistence (including measurement method and duration), and Adverse Events (CTCAE v5.0). Additional variables (immunophenotyping details, genetic markers, and extended cytokine assessments) were extracted to ensure completeness but are not presented in the main manuscript and are available in the supplementary extraction matrix upon request.

### 2.6 Critical appraisal

A formal critical appraisal of individual study quality was not performed, as the primary objective was to map the extent and nature of available evidence rather than to provide pooled effect estimates, consistent with JBI guidance for scoping reviews.

## 3 Results

### 3.1 Selection of sources of evidence

The systematic search across the four electronic databases yielded 2,098 records. After 34 duplicates were removed, 2,064 titles and abstracts were screened, of which 2,002 were excluded. Of the 62 reports sought for retrieval, 43 could not be accessed (69.4%): 38 were conference abstracts or poster presentations without an associated full-text publication, and 5 were restricted-access reports for which inter-library loan and direct author contact were unsuccessful. Notably, one of these posters (Sun et al., 2025), describing a non-viral CAR-T trial in malignant pleural mesothelioma, was excluded under the predefined criterion (ii) because complete peer-reviewed individual patient data were not available. Nineteen full-text reports were therefore assessed for eligibility.

**Figure 1:**
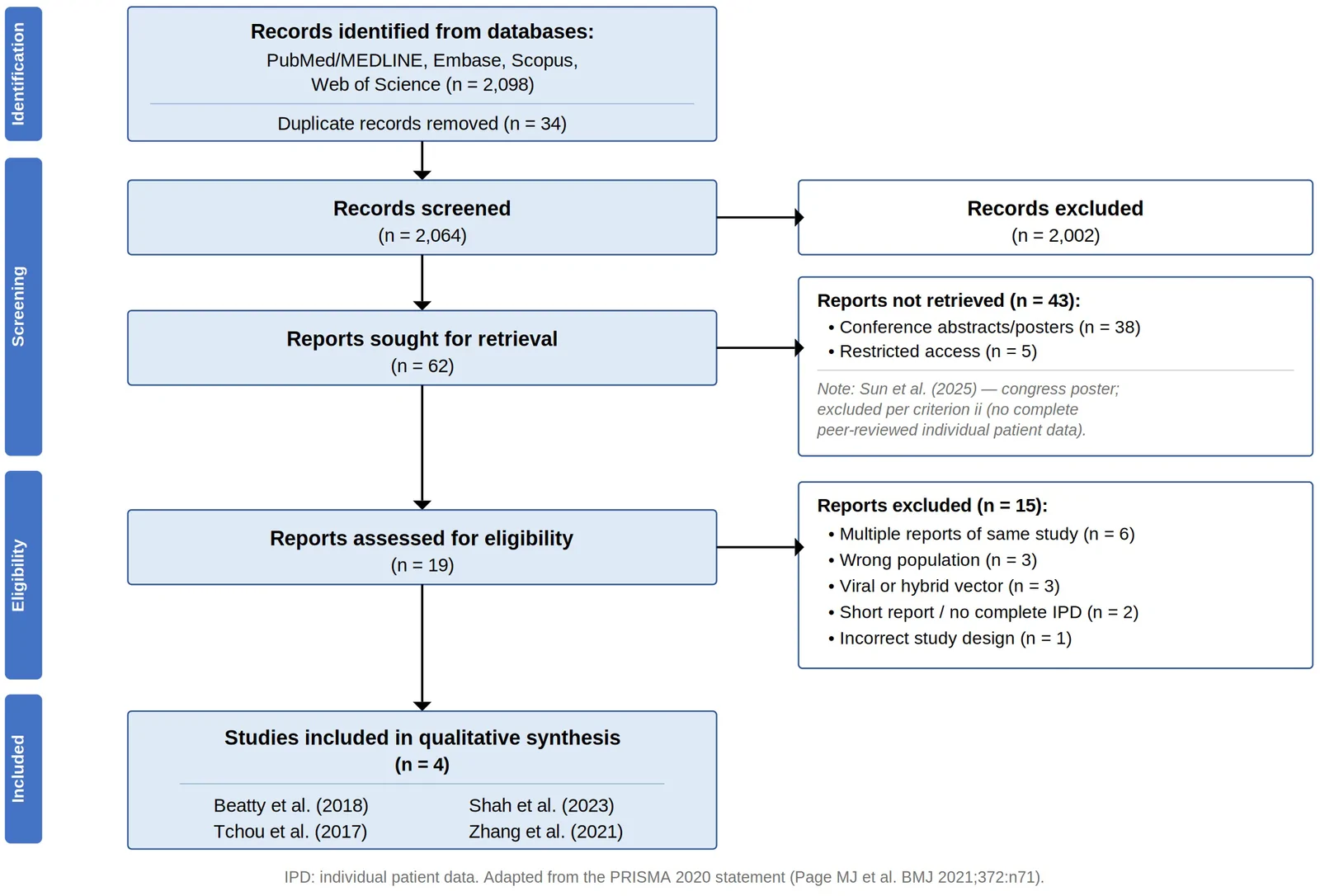
PRISMA 2020 flow diagram of the study selection process. Records identified from four electronic databases (n=2,098). After duplicate removal (n=34), 2,064 records were screened at title/abstract level, excluding 2,002 records. Of 62 reports sought for retrieval, 43 were not retrieved (conference abstracts/posters n=38; restricted access n=5). Nineteen full-text reports were assessed for eligibility; 15 were excluded (multiple reports of same study n=6; wrong population n=3; viral/hybrid vectors n=3; short reports without complete IPD n=2; incorrect study design n=1). Four studies were included in the qualitative synthesis.

Following full-text review, 15 reports were excluded: multiple reports of the same study (n=6), wrong patient population (n=3), use of viral or hybrid vector delivery (n=3), short reports or abstracts lacking complete individual patient data (n=2), and incorrect study design (n=1). Four strictly non-viral clinical studies ultimately met all eligibility criteria and were included in the qualitative synthesis. The complete selection process is summarized in the PRISMA flow diagram (**Figure 1**) (12). Of note, several reports using the Sleeping Beauty transposon system were identified but did not meet the solid-tumor inclusion criterion, as they were conducted exclusively in hematological malignancies.

### 3.2 Characteristics of included studies and patients

The four included studies were published between 2017 and 2023 and together comprised 28 patients with advanced solid tumors treated with non-viral CAR-T cell therapies (13–16). Two non-viral gene delivery platforms were identified across these studies. mRNA electroporation was used in three studies (19 patients) (13–15), whereas the piggyBac transposon system was used in a single study (9 patients) (16). The target antigens were mesothelin (MSLN), c-Met, and the epidermal growth factor receptor (EGFR), and all four studies used autologous T cells as the source for CAR engineering. The principal technical characteristics distinguishing the two platforms — including delivery mechanism, CAR expression duration, cargo capacity, and manufacturing timeline — are summarized in **Table 1**. The individual characteristics and reported outcomes of all 28 patients are detailed in **Table 2**.

**Table 1:** Comparative characteristics of mRNA electroporation and piggyBac transposon platforms for non-viral CAR-T cell engineering.

Characteristic	mRNA electroporation	piggyBac transposon
Delivery mechanism	Transient mRNA transfection via electrical pulses	Stable genomic integration via transposase enzyme
CAR expression duration	Transient (< 7 days)	Sustained (weeks)
Persistence in peripheral blood	Generally undetectable by day +25 post-infusion	Detectable at approximately 28 days (Zhang et al. (16))
Target antigen (included studies)	Mesothelin, c-Met	EGFR
Cargo capacity	Limited (~4 kb)	Larger (~10 kb), enabling additional therapeutic genes
Manufacturing speed	Rapid (days)	Slower (~2 weeks)
*In vivo* CAR expression	Self-limiting, transient	Constitutive, sustained
Key advantage	Rapid iteration; inherently self-limiting expression; lower manufacturing burden	Potential for more durable persistence and response
Key limitation	Transient duration may be insufficient to overcome the solid-tumor microenvironment	Limited clinical evidence to date; small sample size for safety assessment

The clinical evidence for mRNA and piggyBac platforms derives from distinct patient populations, target antigens, tumor types, and administration routes, precluding direct comparative assessment. Both platforms represent promising but still exploratory approaches requiring further clinical investigation.

**Table 2:** Baseline characteristics of the 28 patients treated with strictly non-viral CAR-T cell therapy across the four included studies.

#	Study (Author)	Country	Year	Age	Sex	Primary histology	Metastatic sites	ECOG	Prior lines	Phase	Lymphodepletion
1	Beatty et al. (13)	USA	2018	NP	NP	Pancreatic adenocarcinoma	Liver, Bone, Peritoneum	1	3	I	None
2	Beatty et al. (13)	USA	2018	NP	NP	Pancreatic adenocarcinoma	Liver	1	2	I	None
3	Beatty et al. (13)	USA	2018	NP	NP	Pancreatic adenocarcinoma	Liver, Lymph nodes	0	2	I	None
4	Beatty et al. (13)	USA	2018	NP	NP	Pancreatic adenocarcinoma	Lymph nodes, Peritoneum	1	3	I	None
5	Beatty et al. (13)	USA	2018	NP	NP	Pancreatic adenocarcinoma	Liver	0	2	I	None
6	Beatty et al. (13)	USA	2018	NP	NP	Pancreatic adenocarcinoma	Liver	1	3	I	None
7	Shah et al. (14)	USA	2023	39	F	Triple-negative breast	Lymph nodes	1	6	I	None
8	Shah et al. (14)	USA	2023	58	F	Triple-negative breast	Lymph nodes	0	5	I	None
9	Shah et al. (14)	USA	2023	62	F	Triple-negative breast	Lymph nodes	0	4	I	None
10	Shah et al. (14)	USA	2023	63	F	Triple-negative breast	Liver, Lymph nodes	0	4	I	None
11	Shah et al. (14)	USA	2023	64	M	Melanoma	Lung, Lymph nodes, Skin/soft tissue	1	6	I	None
12	Shah et al. (14)	USA	2023	64	M	Melanoma	Liver, Lung, Lymph nodes, Skin/soft tissue	1	3	I	None
13	Shah et al. (14)	USA	2023	58	F	Triple-negative breast	Liver, Lung, Lymph nodes	0	2	I	None
14	Tchou et al. (15)	USA	2017	44	F	Triple-negative breast	Lung, Lymph nodes	0	3	0	None
15	Tchou et al. (15)	USA	2017	57	F	Triple-negative breast	Lung, Lymph nodes	0	3	0	None
16	Tchou et al. (15)	USA	2017	58	F	Breast, ER-positive	Lung	1	2	0	None
17	Tchou et al. (15)	USA	2017	64	F	Triple-negative breast	Lymph nodes	1	4	0	None
18	Tchou et al. (15)	USA	2017	56	F	Breast, ER-positive	Lymph nodes	0	2	0	None
19	Tchou et al. (15)	USA	2017	60	F	Triple-negative breast	Lung	1	3	0	None
20	Zhang et al. (16)	China	2021	47	F	NSCLC, adenocarcinoma	Pleura, Lymph nodes	NP	1	I	None
21	Zhang et al. (16)	China	2021	72	M	NSCLC, adenocarcinoma	Pleura, Lymph nodes	NP	2	I	None
22	Zhang et al. (16)	China	2021	49	M	NSCLC, adenocarcinoma	Bone, Lymph nodes	NP	2	I	None
23	Zhang et al. (16)	China	2021	73	M	NSCLC, adenocarcinoma	Pleura, Lymph nodes	NP	2	I	None
24	Zhang et al. (16)	China	2021	70	F	NSCLC, adenocarcinoma	Brain	NP	1	I	None
25	Zhang et al. (16)	China	2021	54	M	NSCLC, adenocarcinoma	Bone, Lymph nodes	NP	1	I	None
26	Zhang et al. (16)	China	2021	74	M	NSCLC, squamous	Liver, Lymph nodes	NP	1	I	None
27	Zhang et al. (16)	China	2021	53	F	NSCLC, adenocarcinoma	Bone	NP	2	I	None
28	Zhang et al. (16)	China	2021	56	M	NSCLC, squamous	Adrenal gland, Lymph nodes	NP	1	I	None

Patients are numbered sequentially; the corresponding study reference is indicated in brackets in **Table 3**. Prior lines denotes the number of previous lines of systemic therapy. For Zhang et al. (16), individual ECOG performance status was not reported per patient (all patients met an ECOG 0–1 inclusion criterion); NP indicates not published. ECOG, Eastern Cooperative Oncology Group performance status; CNS, central nervous system; NSCLC, non-small cell lung cancer; NP, not published; NR, none reported.

**Figure 2:**
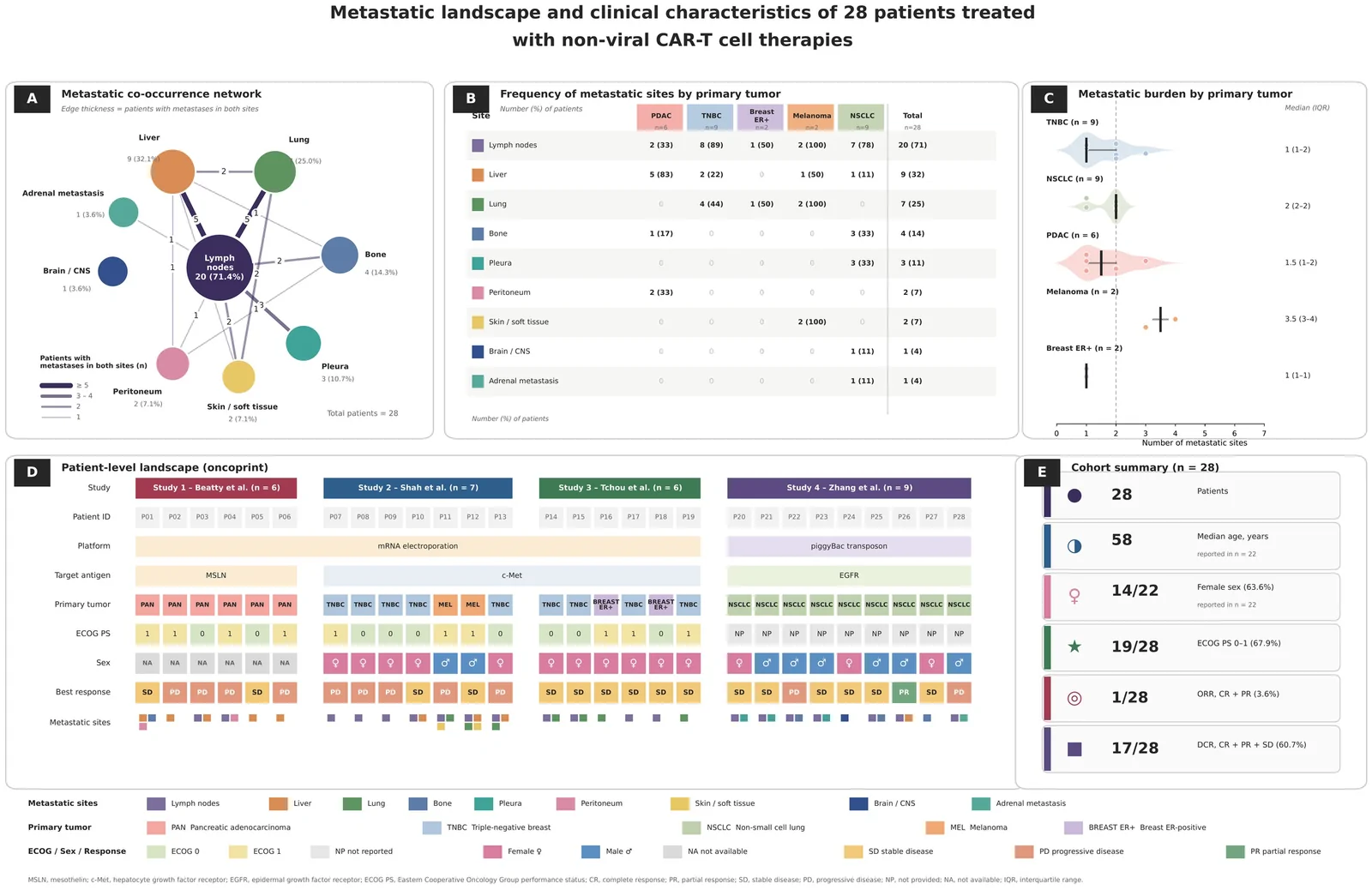
Metastatic landscape and clinical characteristics of the 28 patients treated with non-viral CAR-T cell therapies. **(A)** Metastatic co-occurrence network: each node represents an anatomical metastatic site, node size is proportional to the number of patients with involvement of that site, and edge thickness is proportional to the number of patients with simultaneous involvement of both connected sites. **(B)** Frequency of metastatic sites by primary tumor, expressed as the number (%) of patients within each tumor type. **(C)** Distribution of metastatic burden (number of involved sites per patient) by primary tumor; the vertical bar denotes the median and the horizontal line the interquartile range, with individual patients shown as dots. **(D)** Patient-level oncoprint summarizing, for each of the 28 patients, the source study, non-viral platform, target antigen, primary tumor, ECOG performance status, sex, best overall response (RECIST 1.1), and metastatic sites. **(E)** Aggregate cohort summary. All values are descriptive; percentages are raw proportions (n/N), and no statistical inference was performed. PDAC, pancreatic adenocarcinoma; TNBC, triple-negative breast cancer; NSCLC, non-small cell lung cancer; MSLN, mesothelin; c-Met, hepatocyte growth factor receptor; EGFR, epidermal growth factor receptor; ECOG PS, Eastern Cooperative Oncology Group performance status; SD, stable disease; PD, progressive disease; PR, partial response; CR, complete response; ORR, objective response rate; DCR, disease control rate; IQR, interquartile range; NP, not provided; NA, not available.

The included studies comprised 28 patients with refractory or metastatic solid tumors. Primary histologies were pancreatic adenocarcinoma, triple-negative breast cancer (TNBC), melanoma, and non-small cell lung cancer (NSCLC). The population was heavily pretreated, with the number of prior lines of systemic therapy ranging from one to six across patients. Metastatic involvement was predominantly visceral, most frequently affecting the liver, lungs, and lymph nodes, reflecting the substantial disease burden characteristic of this population. Patient-level details are presented in **Table 2**, and the metastatic landscape and clinical characteristics of the cohort are summarised in **Figure 2**.

### 3.3 Reported outcomes and cellular kinetics

Reported clinical outcomes, described according to RECIST 1.1 criteria, varied across the included studies and their corresponding cellular kinetics profiles; multiple concurrent confounding variables preclude attributing these differences to the delivery platform alone. Across the 19 patients treated with mRNA-electroporated CAR-T cells, cellular persistence was transient, lasting fewer than 7 days, with CAR mRNA reported as undetectable by day +25 after infusion (13–15). No objective responses were documented with this platform (ORR 0/19), although stable disease was reported in 10 of 19 patients, corresponding to a disease control rate (DCR) of 53%. These observations reflect a different patient population, target antigens, administration routes, and study contexts compared to the piggyBac cohort, and therefore direct platform comparison is not appropriate.

In the piggyBac transposon group (n=9), stable genomic integration was associated with a longer reported cellular persistence of approximately 28 days in peripheral blood (16). Within this group, stable disease or better was reported in 7 of 9 patients (DCR 78%), and the only objective response documented across the entire non-viral evidence base — a partial response — occurred in this group (ORR 1/9). Importantly, this cohort differed from the mRNA-treated patients in target antigen (EGFR versus mesothelin/c-Met), primary tumor type (non-small cell lung cancer versus pancreatic, breast, and melanoma), geographic setting, and route of administration. These multiple simultaneous differences preclude attribution of the observed outcome difference to the delivery platform alone, and the finding should be interpreted as descriptive and hypothesis-generating rather than comparative evidence of platform superiority.

### 3.4 Reported adverse events

The adverse events described across both non-viral platforms were limited in severity within this early-phase evidence base. Across the 28 patients included in the identified studies, no Grade ≥3 cytokine release syndrome (CRS) or Grade ≥3 immune effector cell-associated neurotoxicity syndrome (ICANS) was reported, and none of the included publications described a need for tocilizumab or systemic corticosteroids (13–16). Patient-level adverse events are presented in **Table 3**.

**Table 3:** Non-viral delivery platform, target antigen, route of administration, and reported outcomes for the 28 patients across the four included studies.

#	Study (Author)	Platform	Target antigen	Route	Best response	Persistence (days)	CRS	ICANS
1	Beatty et al. (13)	mRNA	MSLN	IV	SD	3	G0	G0
2	Beatty et al. (13)	mRNA	MSLN	IV	PD	3	G0	G0
3	Beatty et al. (13)	mRNA	MSLN	IV	PD	3	G0	G0
4	Beatty et al. (13)	mRNA	MSLN	IV	PD	3	G0	G0
5	Beatty et al. (13)	mRNA	MSLN	IV	SD	3	G0	G0
6	Beatty et al. (13)	mRNA	MSLN	IV	PD	3	G0	G0
7	Shah et al. (14)	mRNA	c-Met	IV	PD	3	G0	G0
8	Shah et al. (14)	mRNA	c-Met	IV	PD	1	G0	G0
9	Shah et al. (14)	mRNA	c-Met	IV	PD	5	G0	G0
10	Shah et al. (14)	mRNA	c-Met	IV	SD	1	G0	G0
11	Shah et al. (14)	mRNA	c-Met	IV	PD	4	G0	G0
12	Shah et al. (14)	mRNA	c-Met	IV	SD	2	G0	G0
13	Shah et al. (14)	mRNA	c-Met	IV	PD	2	G0	G0
14	Tchou et al. (15)	mRNA	c-Met	Intratumoral	SD	3	G0	G0
15	Tchou et al. (15)	mRNA	c-Met	Intratumoral	SD	3	G0	G0
16	Tchou et al. (15)	mRNA	c-Met	Intratumoral	SD	3	G0	G0
17	Tchou et al. (15)	mRNA	c-Met	Intratumoral	SD	3	G0	G0
18	Tchou et al. (15)	mRNA	c-Met	Intratumoral	SD	3	G0	G0
19	Tchou et al. (15)	mRNA	c-Met	Intratumoral	SD	3	G0	G0
20	Zhang et al. (16)	piggyBac	EGFR	IV	SD	28	None	None
21	Zhang et al. (16)	piggyBac	EGFR	IV	SD	28	None	None
22	Zhang et al. (16)	piggyBac	EGFR	IV	PD	NR	None	None
23	Zhang et al. (16)	piggyBac	EGFR	IV	SD	<28	None	None
24	Zhang et al. (16)	piggyBac	EGFR	IV	SD	28	None	None
25	Zhang et al. (16)	piggyBac	EGFR	IV	SD	28	None	None
**26**	**Zhang et al. (16)**	**piggyBac**	**EGFR**	**IV**	**PR**	**<28**	**None**	**None**
27	Zhang et al. (16)	piggyBac	EGFR	IV	SD	28	None	None
28	Zhang et al. (16)	piggyBac	EGFR	IV	PD	<28	None	None

In Zhang et al. (16), cytokine release syndrome and ICANS were not graded per patient; the study reported Grade 1–3 fever in 7 of 9 patients and explicitly documented no serious cytokine release syndrome, hence “None” is shown for both CRS and ICANS in that cohort. For this cohort, persistence reflects detectable peripheral-blood CAR-T cells within the 28-day study window: “28” denotes detection at day 28, “<28” denotes detection after infusion but not at day 28, and “NR” denotes no detectable transgene. mRNA electroporation was used in Beatty et al. (13), Shah et al. (14), and Tchou et al. (15); the piggyBac transposon system was used in Zhang et al. (16). The only documented objective response (partial response) is bolded. Cellular persistence reflects the duration of detectable CAR-T cells in peripheral blood as reported in each source study. It should also be noted that persistence was not measured using a comparable method across platforms: in Beatty et al. (13), Shah et al. (14), and Tchou et al. (15), persistence reflects detection of CAR mRNA transcripts by RT-qPCR, a transient signal expected to decay as unmodified mRNA is degraded; in Zhang et al. (16), persistence reflects detection of the integrated CAR transgene in genomic DNA by qPCR, a signal expected to persist as long as the modified cell survives. Because these assays interrogate fundamentally different molecular targets, cross-platform comparisons of persistence duration are not appropriate. CR, complete response; PR, partial response; SD, stable disease; PD, progressive disease; IV, intravenous; CRS, cytokine release syndrome; ICANS, immune effector cell-associated neurotoxicity syndrome; G, grade (CTCAE v5.0); NP, not published.

## 4 Discussion

The primary objective of this scoping review was to map the clinical landscape of strictly non-viral CAR-T cell therapies in solid tumors. Whereas the conventional viral-vector paradigm is constrained by manufacturing bottlenecks, the risk of insertional mutagenesis, and severe systemic toxicities, the evidence identified across 28 heavily pretreated patients indicates that non-viral platforms have been applied clinically with limited reported adverse events in this early-phase context. The small aggregate sample size and the absence of comparator arms preclude any conclusion regarding comparative performance relative to viral platforms. Notably, the identified evidence raises an exploratory hypothesis that merits prospective investigation: that the degree of *in vivo* cellular persistence may be associated with the pattern of reported clinical outcomes across different non-viral delivery platforms.

### 4.1 Cellular persistence and reported clinical outcomes: mRNA vs. transposon integration

A notable descriptive observation in this review is the difference in reported objective responses and disease control rates between transient mRNA delivery and stable transposon integration. This comparison must be interpreted with considerable caution, because the two platforms differed simultaneously in target antigen (c-Met/MSLN versus EGFR), tumor histology, country of origin, and route of administration — confounding factors that preclude attributing any difference in reported clinical outcomes to the delivery platform alone.

In the piggyBac transposon study (Zhang et al.), stable genomic integration was associated with a longer reported cellular persistence of approximately 28 days, with circulating CAR-T cells detectable in most patients at one month. Within this strictly non-viral evidence base, this study reported the highest disease-control rate (7 of 9 patients) and the only documented objective response across the entire cohort (a partial response sustained beyond 13 months). Although biologically plausible and consistent with preclinical data, this association between prolonged persistence and clinical activity should be regarded as hypothesis-generating, given that it rests on a single objective response and on substantial heterogeneity among the included studies.

### 4.2 Contextualizing findings within the broader CAR-T landscape

Direct comparison with viral vector-based CAR-T platforms in hematological malignancies, such as CD19-directed constructs, is not undertaken in this review. Such cross-indication comparisons are fundamentally constrained by differences in disease biology, target antigen, tumor microenvironment, and manufacturing platform, and would not yield scientifically valid inferences. As discussed in the Limitations section, the early-phase nature of the included trials and the heterogeneity of platforms, target antigens, and patient populations preclude any meaningful comparison between the non-viral solid-tumor evidence synthesized here and published outcomes from viral vector-based platforms in other disease contexts.

### 4.3 Interpreting the mRNA platform findings

The consistent absence of objective responses across all 19 mRNA-treated patients (ORR 0%) warrants explicit discussion. This lack of clinical benefit occurred despite demonstrated CAR-T cell manufacturing, transient systemic expansion, and disease stabilization in a subset. The transient nature of mRNA-delivered CAR expression—lasting fewer than 7 days—may be insufficient to mount sustained antitumor activity against the hostile solid-tumor microenvironment. Alternatively, factors related to patient population (heavily pretreated, advanced disease), target antigen expression levels (mesothelin/c-Met), or route of administration (intravenous vs. intratumoral) may have limited clinical efficacy independent of platform choice. These questions remain unanswered in the current limited evidence base.

### 4.4 Overcoming the solid tumor microenvironment

The detailed mapping of metastatic sites (**Table 2**) provides relevant context for interpreting the reported clinical outcomes. The included patients presented with a substantial visceral disease burden, predominantly affecting the liver, lungs, and lymph nodes. The solid-tumor microenvironment in these organs is characterized by dense physical stroma, hypoxia, and high concentrations of inhibitory cytokines, which rapidly induce T-cell exhaustion (17, 18). On the basis of preclinical models and the limited clinical data available, stable non-viral integration may enhance CAR T-cell fitness and persistence (19), consistent with the hypothesis outlined above.

### 4.5 Reported adverse events across included studies

Across all included studies, the adverse events described in the identified reports were notable for their limited severity. In this 28-patient evidence base, no Grade ≥3 CRS, no Grade ≥3 ICANS, and no toxicities requiring tocilizumab or systemic corticosteroids were reported in any of the included publications. These findings cannot be directly contrasted with the adverse-event profile of viral CAR-T products, given the sample-size and design limitations already noted (20). These observations highlight a gap in the evidence base that warrants prospective investigation of combination strategies and repeated dosing regimens in adequately powered clinical trials.

### 4.6 Limitations and future directions

This scoping review has several limitations. First, the deliberate exclusion of hybrid viral/non-viral models reduced the eligible literature and yielded a small aggregate sample (N = 28). Second, the high proportion of non-retrieved reports (43 of 62; 69.4%) — mostly conference abstracts and posters without full-text publications — may have limited the comprehensiveness of the synthesis. Third, in keeping with JBI guidance for scoping reviews, no formal critical appraisal of study quality was undertaken; the methodological characteristics and principal design limitations of each included study are nonetheless summarized descriptively in **Table 4**. Fourth, the early-phase nature of the included trials (Phase 0/I) means that the primary endpoints were oriented towards feasibility and dose-escalation rather than definitive clinical outcomes. Fifth, heterogeneous outcome reporting precluded any formal meta-analysis. Sixth, the route of administration differed across the mRNA studies (intratumoral injection in Tchou et al. versus intravenous infusion in Beatty et al. and Shah et al.), which limits comparison of peripheral-blood cellular persistence. Finally, the limited follow-up (maximum 13 months) constrains any conclusion regarding the long-term durability of responses.

**Table 4:** Summary of methodological characteristics and key limitations of the four included studies.

#	Study (Author)	Phase	n	Design	Key limitations
1	Beatty et al. (13)	Phase I	6	Open-label pilot	Very small sample size; heterogeneous prior treatment history; single institution; no dose-escalation cohorts; limited long-term follow-up; no comparator or control arm
2	Shah et al. (14)	Phase I	7	Open-label pilot	Extremely small sample; mixed tumor types (melanoma, TNBC); no formal dose-response analysis; limited long-term safety data; single institution
3	Tchou et al. (15)	Phase 0	6	Open-label proof-of-concept	Phase 0 design (very early stage); intratumoral route limits generalizability of systemic persistence data; minimal efficacy endpoints; small cohort; no dose-escalation
4	Zhang et al. (16)	Phase I	9	Open-label trial	Single-center cohort; limited follow-up duration (maximum 13 months); early-phase population; NSCLC only; no long-term durability assessment

All four studies were early-phase (Phase 0/I) trials with limited sample sizes and heterogeneous patient populations, precluding definitive conclusions regarding platform efficacy or comparative safety. Primary endpoints across all studies were feasibility and safety assessment rather than efficacy. These limitations are inherent to the early-phase nature of non-viral CAR-T clinical translation and do not diminish the scientific value of these trials, which serve to establish feasibility and generate hypotheses for future, adequately powered investigation.

### 4.7 Publication bias and evidence base limitations

This scoping review identified a high proportion of non-retrieved reports (43 of 62; 69.4%), predominantly conference abstracts and poster presentations without associated full-text publications. It is plausible that negative or inconclusive non-viral CAR-T trials remain unpublished or are disseminated only through preliminary conference presentations, creating potential publication bias in this evidence base. These factors compound the sample-size and design limitations already noted, reinforcing the exploratory, hypothesis-generating nature of this evidence base and the need for adequately powered prospective trials.

### 4.8 Impact of non-retrieved reports on evidence completeness

The high proportion of non-retrieved records (69.4%) represents a substantial limitation with important implications. If these 43 unretrieved reports included clinical trials with negative findings or unfavorable safety profiles, the current evidence base would be skewed toward positive or neutral outcomes. The nascent stage of the field and the predominance of conference abstracts among non-retrieved records suggest that many ongoing or preliminary studies have not yet been published in peer-reviewed venues. Consequently, the true scope and heterogeneity of non-viral CAR-T clinical investigations may be substantially broader than reflected in the four included studies, and the current body of evidence may not fully represent the field’s challenges and failures alongside successes.

### 4.9 Emerging therapeutic combinations

Recent literature suggests that combination strategies pairing CAR-T cells with checkpoint inhibitors or other immunomodulatory agents may enhance clinical efficacy in solid tumors. Notably, none of the four included studies evaluated concurrent combination regimens; all trials employed single-agent CAR-T approaches. This represents a significant gap in the non-viral CAR-T evidence base, as synergistic combinations remain unexplored at the clinical level in strictly non-viral settings.

Several clinical trials are currently investigating mRNA and transposon-based CAR-T approaches in solid tumors, including trials evaluating repeated dosing strategies and combination approaches with checkpoint inhibitors. Additionally, emerging technologies including AI-guided CAR design, advanced nanomedicine-assisted delivery systems, and metabolic reprogramming strategies during CAR-T manufacturing show promise in preclinical models and early clinical translation for enhancing persistence and efficacy in hostile tumor microenvironments (24). Future clinical trials in solid-tumor oncology may consider the prospective evaluation of stable non-viral integration methods, such as transposon systems, ideally in head-to-head designs that control for target antigen, tumor histology, and lymphodepletion regimen to permit valid comparisons between platforms. In parallel, optimizing lymphodepletion protocols and exploring rational combinations — for example, with immune checkpoint inhibitors — will be essential to inform the clinical development of persistent non-viral CAR-T platforms in solid-tumor oncology (21).

### 4.10 Emerging non-viral approaches and cross-disciplinary context

Beyond the platforms assessed in this review, *in vivo* CAR-T cell engineering using lipid nanoparticle-mediated mRNA delivery represents an orthogonal non-viral approach of considerable scientific interest, in which CAR-encoding mRNA is delivered directly to circulating T cells to generate CAR-T cells within the patient, thereby eliminating the need for leukapheresis and ex vivo manufacturing (22). Although robust preclinical proof-of-concept has been established across tumor models, it is notable that, despite the intensity of investigational activity in this space, clinical evidence in the solid tumor setting remains limited (22). In marked contrast, CAR-T cell therapy has demonstrated striking clinical translation in non-oncological settings: Mackensen et al. reported durable disease remission in patients with treatment-refractory systemic lupus erythematosus following autologous anti-CD19 CAR-T cell infusion (23). Taken together, these observations underscore that the clinical translation of CAR-T cell engineering is, at present, advancing more rapidly in autoimmune disease than in the solid tumor oncology setting, reinforcing the relevance of mapping and characterising the still-nascent clinical evidence base for non-viral solid-tumor applications.

## 5 Conclusions

The clinical translation of strictly non-viral CAR-T cell therapies remains at an early stage in the search for effective cellular immunotherapies in solid tumors. By rigorously excluding hybrid viral/non-viral models, this scoping review identified that, across a limited early-phase evidence base of 28 patients in four published studies, no Grade ≥3 CRS or ICANS was reported in association with any of the strictly non-viral platforms included. The only documented objective response across the included studies was reported in association with the platform describing the longest *in vivo* cellular persistence; whether this association reflects a causal relationship cannot be determined from the available descriptive evidence.

Among the included studies that used transient-expression platforms — specifically mRNA electroporation — the adverse-event profiles described were limited in severity, and disease stabilization was reported in a subset of patients; however, no objective responses were documented in these reports. These descriptive findings support the need for prospective clinical investigation of stable non-viral genomic-integration platforms for solid-tumor CAR-T design, particularly studies with adequate comparator arms and standardized outcome reporting.

Technologies such as the piggyBac transposon system extend cellular persistence and offer the genomic capacity for multiple therapeutic edits, potentially with a distinct safety and logistical profile compared with viral vectors; however, this observation derives from a single nine-patient study and should be regarded as exploratory and hypothesis-generating rather than definitive evidence of platform superiority. As the field advances beyond early-phase safety and dose-escalation studies, rigorous evaluation of stable non-viral integration platforms — potentially in combination with immune checkpoint blockade or optimized lymphodepletion — will be necessary to characterize their clinical applicability in solid-tumor oncology, ideally through adequately powered trials with prespecified comparator arms.

## Data Availability

The original contributions presented in the study are included in the article/**Supplementary Material**. Further inquiries can be directed to the corresponding authors.
