# Supplementary material for: Clinical evidence on non-viral CAR-T cell therapies for solid tumors: a scoping review
Source: Front Immunol. 2026 Aug 20;17:1905685. doi: 10.3389/fimmu.2026.1905685 (PMC13538877; doi:10.3389/fimmu.2026.1905685)
Supplement: Supplementary file 1 [file Table1.docx]

**Preferred Reporting Items for Systematic reviews and Meta-Analyses extension for Scoping Reviews (PRISMA-ScR) Checklist**

***Clinical Evidence on Non-Viral CAR-T Cell Therapies for Solid Tumors: A Scoping Review***

Varón F, Moreno J, Arias-Valderrama O, Baena J. Universidad Icesi / Fundación Valle del Lili, Cali, Colombia.

*Supplementary Material S1 | Corresponding author: faviovaronsuarez@gmail.com*

| **SECTION** | **ITEM** | **PRISMA-ScR CHECKLIST ITEM** | **REPORTED ON PAGE #** |
| --- | --- | --- | --- |
| **TITLE** | | | |
| **Title** | 1 | Identify the report as a scoping review. | Page 1 |
| **ABSTRACT** | | | |
| **Structured summary** | 2 | Provide a structured summary that includes (as applicable): background, objectives, eligibility criteria, sources of evidence, charting methods, results, and conclusions that relate to the review questions and objectives. | Pages 1–2 |
| **INTRODUCTION** | | | |
| **Rationale** | 3 | Describe the rationale for the review in the context of what is already known. Explain why the review questions/objectives lend themselves to a scoping review approach. | Pages 2–3 |
| **Objectives** | 4 | Provide an explicit statement of the questions and objectives being addressed with reference to their key elements (e.g., population or participants, concepts, and context) or other relevant key elements used to conceptualize the review questions and/or objectives. | Page 3 |
| **METHODS** | | | |
| **Protocol and registration** | 5 | Indicate whether a review protocol exists; state if and where it can be accessed (e.g., a Web address); and if available, provide registration information, including the registration number. Protocol registered a priori on the Open Science Framework (OSF). Registration available at: https://doi.org/10.17605/OSF.IO/2TPQS | Page 3 |
| **Eligibility criteria** | 6 | Specify characteristics of the sources of evidence used as eligibility criteria (e.g., years considered, language, and publication status), and provide a rationale. Inclusion: human subjects with solid tumors; strictly non-viral CAR-T platforms (mRNA electroporation or transposon); extractable clinical outcomes (efficacy, safety, cellular kinetics). No language restrictions. Exclusion: preclinical/in vitro studies; reviews, commentaries, congress abstracts, poster presentations; viral or hybrid vectors. | Pages 3–4 |
| **Information sources** | 7 | Describe all information sources in the search (e.g., databases with dates of coverage and contact with authors to identify additional sources), as well as the date the most recent search was executed. PubMed/MEDLINE, Embase, Scopus, Web of Science Core Collection. Search from inception to May 2026. Contact with authors attempted for 5 restricted-access records. | Page 4 |
| **Search** | 8 | Present the full electronic search strategy for at least 1 database, including any limits used, such that it could be repeated. MeSH and free-text keywords: "Chimeric Antigen Receptor T-Cells", "non-viral", "mRNA", "electroporation", "transposon", "piggyBac", "gene delivery", "solid tumor". No language limits. Complete Boolean strings per database in Supplementary Material S2. | Page 4 Suppl. S2 |
| **Selection of sources of evidence** | 9 | State the process for selecting sources of evidence (i.e., screening and eligibility) included in the scoping review. Two-stage screening via Rayyan: (1) title/abstract by two independent reviewers; (2) full-text assessment. Discrepancies resolved by consensus or senior methodologist. | Page 4 |
| **Data charting process** | 10 | Describe the methods of charting data from the included sources of evidence (e.g., calibrated forms or forms that have been tested by the team before their use, and whether data charting was done independently or in duplicate) and any processes for obtaining and confirming data from investigators. Pre-structured instrument of 53 clinical and technical variables applied independently by two reviewers at the individual patient level (IPD). Discrepancies resolved by consensus. | Pages 4–5 |
| **Data items** | 11 | List and define all variables for which data were sought and any assumptions and simplifications made. Variables included: study characteristics, demographics, prior therapy lines, histology, metastatic sites (binary mapping), lymphodepletion regimen, delivery platform, best global response (RECIST 1.1), cellular persistence, and severe adverse events (CTCAE v5.0). 53 total variables. | Page 5 |
| **Critical appraisal of individual sources of evidence** | 12 | If done, provide a rationale for conducting a critical appraisal of included sources of evidence; describe the methods used and how this information was used in any data synthesis (if appropriate). Formal critical appraisal was not performed, consistent with JBI guidance for scoping reviews, whose primary objective is to map the extent and nature of evidence rather than assess study quality. | Page 5 |
| **Synthesis of results** | 13 | Describe the methods of handling and summarizing the data that were charted. Descriptive qualitative synthesis. Proportions reported with 95% Wilson score confidence intervals. No pooled effect estimates or meta-analysis performed. | Page 5 |
| **RESULTS** | | | |
| **Selection of sources of evidence** | 14 | Give numbers of sources of evidence screened, assessed for eligibility, and included in the review, with reasons for exclusions at each stage, ideally using a flow diagram. Identified: 2,098 → after deduplication: 2,064 → screened: 2,064 → excluded title/abstract: 2,002 → sought retrieval: 62 → not retrieved: 43 → assessed eligibility: 19 → excluded full-text: 15 → included: 4. PRISMA 2020 flow diagram provided as Figure 1. | Pages 5–6 Figure 1 |
| **Characteristics of sources of evidence** | 15 | For each source of evidence, present characteristics for which data were charted and provide the citations. Beatty et al. (2018), Shah et al. (2023), Tchou et al. (2017), Zhang et al. (2021). Characteristics presented in Table 1 (study design, platform, antigen, cell source) and Table 2 (demographics, histology, prior therapy). | Pages 6–7 Tables 1–2 |
| **Critical appraisal within sources of evidence** | 16 | If done, present data on critical appraisal of included sources of evidence (see item 12). Not applicable. Formal critical appraisal was not performed (see item 12). | Page 5 (N/A) |
| **Results of individual sources of evidence** | 17 | For each included source of evidence, present the relevant data that were charted that relate to the review questions and objectives. Individual study results presented in Table 4 (clinical efficacy and cellular persistence: ORR, DCR, PD with 95% CI by platform and route) and Table 5 (safety: CRS, ICANS, cytopenias G≥3, tocilizumab/steroid use). | Pages 7–8 Tables 4–5 |
| **Synthesis of results** | 18 | Summarize and/or present the charting results as they relate to the review questions and objectives. Descriptive synthesis presented in Results sections: Efficacy and Cellular Kinetics (mRNA: ORR 0%, DCR 53%; piggyBac: ORR 11%, DCR 78%) and Safety Profile (no Grade ≥3 CRS or ICANS across 28 patients). | Pages 7–9 |
| **DISCUSSION** | | | |
| **Summary of evidence** | 19 | Summarize the main results (including an overview of concepts, themes, and types of evidence available), link to the review questions and objectives, and consider the relevance to key groups. Discussion addresses: (1) persistence-efficacy axis (mRNA vs. piggyBac); (2) tumor microenvironment context; (3) consistent safety signal. All findings framed as hypothesis-generating given small sample size and multiple confounders. | Pages 9–11 |
| **Limitations** | 20 | Discuss the limitations of the scoping review process. Seven limitations discussed: (1) narrow eligibility excluding hybrid models; (2) high non-retrieval rate (69.4%); (3) no formal critical appraisal; (4) early-phase trials with safety/feasibility endpoints; (5) heterogeneous outcome reporting; (6) differing routes of administration; (7) limited follow-up (max 13 months). | Pages 11–12 |
| **Conclusions** | 21 | Provide a general interpretation of the results with respect to the review questions and objectives, as well as potential implications and/or next steps. Non-viral CAR-T platforms show a preliminary favorable safety profile. The only objective response was associated with the stable integration platform (piggyBac). Results support further investigation of stable non-viral platforms, ideally in head-to-head designs controlling for antigen, histology, and lymphodepletion. | Page 12 |
| **FUNDING** | | | |
| **Funding** | 22 | Describe sources of funding for the included sources of evidence, as well as sources of funding for the scoping review. Describe the role of the funders of the scoping review. This scoping review received no specific funding from public, commercial, or not-for-profit sectors. Funding sources for included studies are described in their respective publications. | Page 12 |

*JBI = Joanna Briggs Institute; PRISMA-ScR = Preferred Reporting Items for Systematic reviews and Meta-Analyses extension for Scoping Reviews; IPD = individual patient data; OSF = Open Science Framework; RECIST = Response Evaluation Criteria in Solid Tumors; CTCAE = Common Terminology Criteria for Adverse Events; CRS = cytokine release syndrome; ICANS = immune effector cell-associated neurotoxicity syndrome.*

*From: Tricco AC, Lillie E, Zarin W, et al. PRISMA Extension for Scoping Reviews (PRISMA-ScR): Checklist and Explanation. Ann Intern Med. 2018;169:467–473. doi: 10.7326/M18-0850.*
