# Supplementary material for: Clinical evidence on non-viral CAR-T cell therapies for solid tumors: a scoping review
Source: Front Immunol. 2026 Aug 20;17:1905685. doi: 10.3389/fimmu.2026.1905685 (PMC13538877; doi:10.3389/fimmu.2026.1905685)
Supplement: Supplementary file 2 [file Table2.docx]

# Supplementary Material S2 | Database Search Strategies

***Clinical Evidence on Non-Viral CAR-T Cell Therapies for Solid Tumors: A Scoping Review***

Varón F, Moreno J, Arias-Valderrama O, Baena J. Universidad Icesi / Fundación Valle del Lili, Cali, Colombia.

*Corresponding author: faviovaronsuarez@gmail.com.*

The systematic search was conducted across four electronic databases from database inception to 15 May 2026, with no language restrictions. The search combined Medical Subject Headings (MeSH/Emtree) and free-text terms across three concept blocks: (1) chimeric antigen receptor T cells, (2) strictly non-viral gene-delivery methods, and (3) solid tumors, combined with the Boolean operator AND. The complete strategy for each database is reproduced below. A total of 2,098 records were identified; after removal of 34 duplicates, 2,064 records were screened. The study selection process is summarized in the PRISMA 2020 flow diagram (Figure 1).

## 1. PubMed / MEDLINE (NCBI)

*Coverage: inception to 15 May 2026 | Date of last search: 15 May 2026 | Interface: NCBI PubMed*

```
#1  "Receptors, Chimeric Antigen"[MeSH] OR "chimeric antigen receptor*"[tiab]
    OR "CAR-T"[tiab] OR "CAR T cell*"[tiab] OR "CAR-T cell*"[tiab]

#2  "non-viral"[tiab] OR "nonviral"[tiab] OR "mRNA"[tiab]
    OR "messenger RNA"[tiab] OR "Electroporation"[MeSH] OR "electroporation"[tiab]
    OR "DNA Transposable Elements"[MeSH] OR "transposon*"[tiab]
    OR "transposase*"[tiab] OR "piggyBac"[tiab] OR "Sleeping Beauty"[tiab]
    OR "gene delivery"[tiab] OR "gene transfer"[tiab]

#3  "Neoplasms"[MeSH] OR "solid tumor*"[tiab] OR "solid tumour*"[tiab]
    OR "solid cancer*"[tiab] OR "carcinoma*"[tiab] OR "sarcoma*"[tiab]
    OR "melanoma*"[tiab]

#4  #1 AND #2 AND #3
```

## 2. Embase (Elsevier)

*Coverage: inception to 15 May 2026 | Date of last search: 15 May 2026 | Interface: Embase.com*

```
#1  'chimeric antigen receptor'/exp OR 'chimeric antigen receptor t cell'/exp
    OR 'chimeric antigen receptor*':ti,ab OR 'CAR-T':ti,ab OR 'CAR T cell*':ti,ab

#2  'nonviral gene delivery system'/exp OR 'non-viral':ti,ab OR 'nonviral':ti,ab
    OR 'mRNA':ti,ab OR 'messenger RNA'/exp OR 'electroporation'/exp
    OR 'electroporation':ti,ab OR 'transposon'/exp OR 'transposon*':ti,ab
    OR 'piggyBac':ti,ab OR 'sleeping beauty':ti,ab OR 'gene delivery'/exp
    OR 'gene transfer':ti,ab

#3  'solid tumor'/exp OR 'solid tumor*':ti,ab OR 'solid tumour*':ti,ab
    OR 'neoplasm'/exp

#4  #1 AND #2 AND #3
```

## 3. Scopus (Elsevier)

*Coverage: inception to 15 May 2026 | Date of last search: 15 May 2026 | Field: TITLE-ABS-KEY*

```
TITLE-ABS-KEY ( "chimeric antigen receptor*" OR "CAR-T"
                OR "CAR T cell*" )
AND TITLE-ABS-KEY ( "non-viral" OR "nonviral" OR "mRNA"
                    OR "messenger RNA" OR "electroporation"
                    OR "transposon*" OR "piggyBac" OR "sleeping beauty"
                    OR "gene delivery" OR "gene transfer" )
AND TITLE-ABS-KEY ( "solid tumor*" OR "solid tumour*"
                    OR "solid cancer*" OR neoplasm* )
```

## 4. Web of Science Core Collection (Clarivate)

*Coverage: inception to 15 May 2026 | Date of last search: 15 May 2026 | Field tag: TS (Topic)*

```
TS = ( "chimeric antigen receptor*" OR "CAR-T" OR "CAR T cell*" )
AND TS = ( "non-viral" OR "nonviral" OR "mRNA" OR "messenger RNA"
           OR "electroporation" OR "transposon*" OR "piggyBac"
           OR "sleeping beauty" OR "gene delivery" OR "gene transfer" )
AND TS = ( "solid tumor*" OR "solid tumour*" OR "solid cancer*"
           OR "neoplasm*" )
```

**Note on screening.** Records were exported to the Rayyan systematic review platform, where two reviewers independently screened titles and abstracts, followed by full-text assessment. Discrepancies were resolved by consensus or by a senior methodologist.
